# Conjunctival leiomyosarcoma

**DOI:** 10.1016/j.ajoc.2022.101580

**Published:** 2022-05-11

**Authors:** Aluisio Rosa Gameiro Filho, Ana Marisa Pires Castello Branco, Moacyr Pezati Rigueiro, Melina Correia Morales, Rubens N. Belfort

**Affiliations:** a Ophthalmology and Visual Sciences Department, Universidade Federal de São Paulo (Unifesp – EPM), R. Botucatu, 822 - Vila Clementino, São Paulo, SP, 04023-062, Brazil; b Pathology Department, Universidade Federal de São Paulo (Unifesp –EPM), R. Botucatu, 720 - Vila Clementino, São Paulo, SP, 04023-062, Brazil

**Keywords:** Leiomyosarcoma, Conjunctiva, Eye, Tumor, Sarcoma, Soft tissue tumours

## Abstract

**Purpose:**

Leiomyosarcoma (LMS) is a mesenchymal neoplasm with smooth muscle differentiation, being considered one of the most common soft tissue sarcomas. However, it rarely affects the eye, and when it does, it is usually located in the orbit, being extremely rare in the conjunctiva.

**Observations:**

We report a case of a 45 years old male patient, with a recurrent rapid growing conjunctival mass on the temporal limbus of his left eye, which was excised, and the anatomopathological report was suggestive of a grade 1 leiomyosarcoma. Since the lesion was recurrent, we decided to perform an extended enucleation for treating this condition. Nevertheless, the patient is being followed up to 30 months, with systemic metastasis screening, showing no other lesions or recurrences.

**Conclusions and importance:**

Conjunctival leiomyosarcoma is an extremely rare ocular tumor, which can be clinically indistinguishable from other conditions such as squamous cell carcinoma, so, biopsy is essential. Albeit there is no standard treatment, complete surgical removal with safety margins is mandatory.

## 1 Introduction

Leiomyosarcomas (LMS) are mesenchymal neoplasms with smooth muscle differentiation. It accounts for up to 25% of all soft tissue sarcomas,^1^ and it is responsible for less than 1% of all malignancies.^2^ It can arise from several anatomic sites, the most common being the abdominal-pelvic region, large blood vessels, and uterus.^1^ Leiomyosarcoma of the eye and adnexa is considered very rare, even more, when affecting the conjunctiva. We report a case of a patient with a primary conjunctival leiomyosarcoma.

## 2 Case report

A 45 years old male patient was referred to the Ocular Oncology ambulatory of the Department of Ophthalmology and Visual Sciences at Escola Paulista de Medicina, due to a rapidly growing mass in the temporal conjunctiva of his left eye, associated with redness and irritation. He had a history of excision of a similar lesion in the same topography, performed in another hospital one year before, with an anatomopathological report suggestive of leiomyosarcoma.

**Fig. 1:**
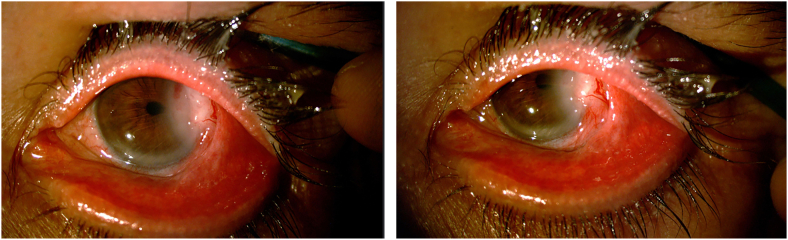
Biomicroscopy showing a whitish elevated and vascularized lesion in the temporal limbus and temporal conjuntiva. An infiltrative aspect is noticeable, as well as extension to the cornea, which presents folds in the perilesional region. A retraction of the tarsal conjunctiva can also be seen.

On ophthalmological examination, he presented a vascularized whitish lesion in the temporal limbus, with an infiltrative aspect. The lesion extended to the cornea (creating folds in the perilesional region, with an aspect of deep corneal invasion), temporal conjunctiva, and causing retraction of the tarsal conjunctiva (Fig. 1). Slides of the previous biopsy were requested and reviewed, showing atypical spindle cell proliferation. Immunohistochemistry analysis was performed revealing positivity to Vimentin, Calponin, and Desmin, which is compatible with LMS. Patient underwent a systemic screening for other sites of tumours, with negative results.

**Fig. 2:**
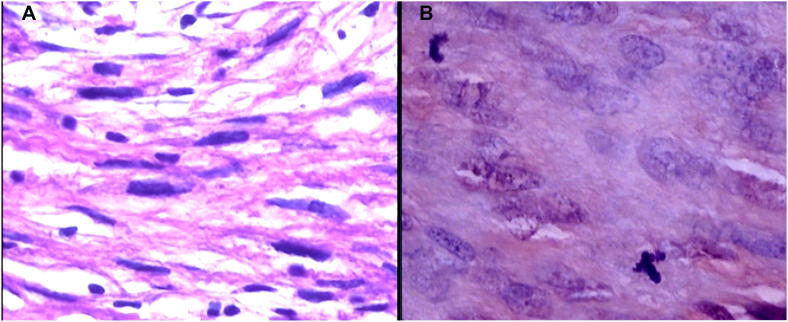
Histopathological analysis showing atypical fusiform cells and nuclear pleomorphism (A). Atypical mitosis can be seen on image B (Hematoxylin-Eosin staining 400x).

**Fig. 3:**
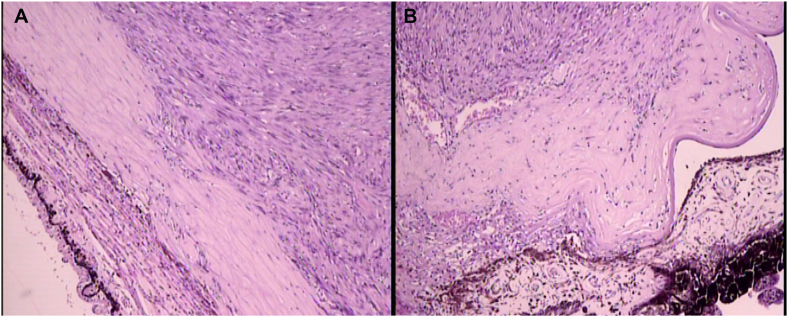
Histopathological analysis showing scleral invasion (image A, H-E 100x) and corneal invasion (image B, H-E 40x).

**Fig. 4:**
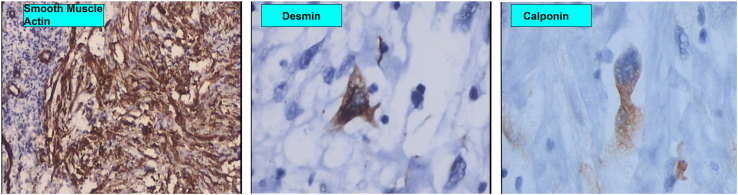
Immunohistochemistry analysis shows staining for A = Smooth muscle actin (200x). B= Calponin (400x). C = Desmin (400x).

We decided to perform an extended enucleation, mostly because the infiltrative aspect of the lesion and also due to the fact that the lesions was previously biopsed, and the material was sent to further analysis. Biopsy showed fascicles of spindle cells with eosinophilic cytoplasm and areas of atypia. Necrosis was absent; however, areas of haemorrhage were present. Up to 2 mitosis per high-power field (HFP) was noticed, some of them being atypical. Scleral and corneal deep stroma invasion was present. The chamber angle was not invaded. Immunohistochemistry was positive for Vimentin, Calponin, Desmin, Caldesmon, CD68, AE1/AE3 (focal in rare cells), and SMA (Fig. 2, Fig. 3, Fig. 4) confirming the recurrence of a grade 1 LMS, according to the *Fédération National des Centres de Lutte Contre Le Cancer (*FNCLCC).

The patient is being followed by a clinical oncologist, with imaging exams of the central nervous system (CNS), chest, and abdomen for 30 months without recurrence.

## 3 Discussion

Leiomyosarcomas are malignant tumours showing smooth muscle differentiation, with multiple genomic aberrations. It is characterised by non-recurrent structural and copy number alterations.^3^ The underlying genetic mechanism is not completely understood, however, there is evidence that up to 90% of LMS patients present loss of RB1 function. Furthermore, 40–50% of cases have shown a loss of chromosome 10q, which encompass PTEN, another tumor suppressor gene.^3^

Several orbital leiomyosarcomas have been reported in the literature, as well as metastatic LMS to the orbit and choroid. However, to the best of our knowledge, only 12 cases of primary conjunctival LMS have been previously reported.^2^, ^3^, ^4^, ^5^, ^6^, ^7^, ^8^, ^9^, ^10^, ^11^, ^12^ In a series of 1.643 conjunctival tumours from Wills Eye, there was no description of LMS,^13^ also, in a review of 2455 conjunctival specimens from John Hopkins Eye Pathology Laboratory, no LMS was diagnosed.^14^

The heterogeneous clinical appearance of LMS makes it difficult to distinguish among several other conjunctival conditions such as squamous carcinoma, lymphomas, and amelanotic melanoma. Other possible differential diagnosis would be myofibroma, leiomyoma and nodular fascitis. For that reason, a biopsy is mandatory for the correct diagnosis. The typical histological pattern of LMS is characterised by intersecting sharply marginated fascicles of spindle cells with eosinophilic cytoplasm and elongated hyperchromatic nuclei.^15^ Focal pleomorphism may occur, and occasionally focal storiform or hemagioperycitoma-like arrangement may be seen.^16^

Immunohistochemistry analysis of the lesions usually shows Vimentin, Desmin, Calponin, and Smooth Muscle Actine (SMA) reactivity. AE1/AE3 anti-cytokeratin antibodies can also be positive, due to the anomalous presence of keratin in these tumours.

The overall incidence of LMS increases with age, as in soft tissue sarcomas in general, peaking in the seventh decade of life.^1^ Regarding specifically conjunctival LMS, this seems not to be true. The mean age of all previously reported cases is 52.33 years old (ranging from 20 to 81). Five of these patients had less than 50 years old at the moment of diagnosis.

Regarding the location of the tumor, De Groot^2^ observed that all the lesions described in the literature were located at the limbus. She proposed that conjunctival LMS could be originated from pluripotent steam cells located at the corneoscleral limbus. In this case, the lesion was located in the temporal limbus and within the palpebral aperture.

Some risk factors were previously proposed for LMS. The most important is Epstein-Barr virus (EBV) infection, mainly in the setting of immunosuppression such as acquired immunodeficiency syndrome (AIDS), and immunosuppressants or steroids use.^4^ Other risk factors include radiotherapy and retinoblastoma (RB). Patients with hereditary RB have a cumulative risk of 13.1% for developing any soft tissue sarcoma as secondary malignancy.^1^ In our case, the patient refused to be tested for HIV and EBV.

LMS is considered an aggressive type of tumor, capable of hematogenous dissemination (with a predilection to lungs, liver, and brain), local recurrence, and metastasis (with a reported overall survival of 69% and 60% at 5 and 10 years respectively).^16^ However, in conjunctival cases, it seems that the tumor never invades the sclera in the absence of a previous surgery,^2^ and tend to be less aggressive than LMS on other anatomical sites.

Surgical resection with negative margins is the preferred treatment for LMS.^15^ Radiotherapy can achieve local control and decrease local recurrences, albeit, it does not improve overall survival.^15^ When curative surgery is not possible, systemic treatment with Doxorubicin, Ifosfamine, and Gemcitabine can be done. Since there are only a few cases of conjunctival LMS, there is no standardised treatment for this condition. For that reason, the recommendations for other sites are used for the treatment of conjunctival LMS. According to the literature, six cases of conjunctival LMS underwent aggressive surgeries, such as enucleation, evisceration, or exenteration. However, because conjunctival LMS seems to be less aggressive, a globe-sparring procedure can be an option if only bulbar conjunctiva is involved. In these cases, adjunctive brachytherapy must be performed if margins are not clear. In our case, we decided to perform an extended enucleation because the patient had signs of invasion and a history of previous biopsies.

## 4 Conclusions

Conjunctival LMS is a rare tumor, which should be suspected in patients with conjunctival tumours that look sub-epithelial. The diagnosis can only be made by histopathological and immunohistochemistry analysis. Moreover, due to the rarity of this condition, there is no standard treatment. However, the consensus is that a complete surgical removal with safety margins is mandatory. Globe-sparing procedures can be an option if the tumor is restricted to bulbar conjunctiva. In other cases, a more aggressive surgery, such as enucleation or evisceration is necessary, mostly because of the high recurrences rates of LMS.

## Patient consent

Patient has provided written informed consent.

## Funding

No funding or grant support.

## Conflits of interest

There are no conflicts of interest or financial disclosures for any of the authors listed.

## Authorship

All authors attest that they meet the current ICMJE criteria for Authorship.

## Conflicts of interest

We wish to confirm that there are no known conflicts of interest associated with this publication and there has been no significant financial support for this work that could have influenced its outcome.

## Funding

No funding was received for this work.

## Intellectual property

We confirm that we have given due consideration to the protection of intellectual property associated with this work and that there are no impediments to publication, including the timing of publication, with respect to intellectual property. In so doing we confirm that we have followed the regulations of our institutions concerning intellectual property.

## Research ethics

We further confirm that any aspect of the work covered in this manuscript that has involved human patients has been conducted with the ethical approval of all relevant bodies and that such approvals are acknowledged within the manuscript.

## Authorship

All listed authors meet the ICMJE criteria. We attest that all authors contributed significantly to the creation of this manuscript, each having fulfilled criteria as established by the ICMJE.

We confirm that the manuscript has been read and approved by all named authors.

We confirm that the order of authors listed in the manuscript has been approved by all named authors.

## Contact with the editorial office

The Corresponding Author declared on the title page of the manuscript is:

Conjunctival Leiomyosarcoma.

This author submitted this manuscript using his/her account in EVISE.

We understand that this Corresponding Author is the sole contact for the Editorial process (including EVISE and direct communications with the office). He/she is responsible for communicating with the other authors about progress, submissions of revisions and final approval of proofs.

We confirm that the email address shown below is accessible by the Corresponding Author, is the address to which Corresponding Author's EVISE account is linked, and has been configured to accept email from the editorial office of American Journal of Ophthalmology Case Reports: aluisio.gameiro@unifesp.br.
